# Holocaust Survivor’s stories following the October 7th war in Israel

**DOI:** 10.1093/geroni/igaf122.096

**Published:** 2025-12-31

**Authors:** Yarin Cohen, Maryah Fram, Sue Levkoff

**Affiliations:** University of South Carolina, Columbia, South Carolina, United States; Mary Baldwin University, Columbia, South Carolina, United States; University of South Carolina, Columbia, South Carolina, United States

## Abstract

Holocaust survivors (HS) have often developed narrative identities centered on Israel as both ensuring safety from atrocities and a place where Jewish communities can thrive into the future. The October 7th Hamas attack and subsequent war potentially challenged these resilience fostering narratives. This study explored how HS processed these events and subsequent antisemitism to better understand their meaning-making and support their well-being during late life. Researchers conducted 18 in-depth, semi-structured video interviews with Israeli HS(ages 81-95), analyzing transcripts through thematic and narrative approaches. Findings revealed survivors used their historical narratives as coping tools when processing October 7, drawing guidance from past coping experiences. As a coping mechanism, they shifted between multiple perspectives—viewing events as Jews, as Israelis, and within global-historical contexts. While distinguishing October 7th from the Holocaust, some noted similarities to historical pogroms, with certain images triggering personal Holocaust memories. Many expressed concerns about Israel’s existence, their descendants’ safety, diminished hopes for peace, and anxiety about not living to see crisis resolution due to their age. The resurgence of antisemitism was particularly alarming to many who noted disturbing similarities to pre-Holocaust conditions. Their accounts revealed both psychological distress and remarkable resilience. Participants’ narrative-based coping strategies revealed mechanisms for identity preservation during periods of trauma and challenge. These findings inform how support services might be tailored for older adult HS who continue to face psychological impacts of historical trauma within contexts of ongoing uncertainty, while recognizing them as resilience “experts”, preserving their testimony and deepening understanding of genocide’s psychological impacts.

